# Material Viscoelasticity-Induced Drift of Micro-Accelerometers

**DOI:** 10.3390/ma10091077

**Published:** 2017-09-14

**Authors:** Wu Zhou, Peng Peng, Huijun Yu, Bei Peng, Xiaoping He

**Affiliations:** 1 School of Mechatronics Engineering, University of Electronic Technology and Science of China, Chengdu 611731, China; zhouwu916@uestc.edu.cn (W.Z.); yuhjuestc@126.com (H.Y.); 2 Institute of Electronic Engineering, China Academy of Engineering Physics, Mianyang 621900, China; hexpiee@263.net

**Keywords:** MEMS, adhesive, viscoelasticity, drift, accelerometer

## Abstract

Polymer-based materials are commonly used as an adhesion layer for bonding die chip and substrate in micro-system packaging. Their properties exhibit significant impact on the stability and reliability of micro-devices. The viscoelasticity, one of most important attributes of adhesive materials, is investigated for the first time in this paper to evaluate the long-term drift of micro-accelerometers. The accelerometer was modeled by a finite element (FE) method to emulate the structure deformation and stress development induced by change of adhesive property. Furthermore, the viscoelastic property of the adhesive was obtained by a series of stress–relaxation experiments using dynamic mechanical analysis (DMA). The DMA curve was imported into the FE model to predict the drift of micro-accelerometers over time and temperature. The prediction results verified by experiments showed that the accelerometer experienced output drift due to the development of packaging stress induced by both the thermal mismatch and viscoelastic behaviors of the adhesive. The accelerometers stored at room temperature displayed a continuous drift of zero offset and sensitivity because of the material viscoelasticity. Moreover, the drift level of accelerometers experiencing high temperature load was relatively higher than those of lower temperature in the same period.

## 1. Introduction

In packaging of microelectromechanical systems (MEMS), polymer-based materials, such as epoxy and silicone, are widely used for mounting the die chip on the substrate because of their numerous advantages compared with other joining methods [1,2]. However, this type of adhesive packaging, like other packaging themes, involves residual thermal stress/packaging stress due to the mismatch of coefficients of thermal expansion (CTE) of different structural materials [3]. The stress existing in structures and interfaces forms a stable equilibrium of micro-devices based on deformation compatibility conditions [4]. The formed equilibrium, however, is prone to be upset by the temperature load and/or the shift of material properties, such as elastic module and Poisson’s ratio. The temperature aspects are always related to the thermal effects’ influence on device performance, which has been studied intensively, while the material properties of the polymer-based materials in current studies are often neglected or simply assumed to be linear–elastic [5,6,7,8,9,10]. This assumption could give a relatively accurate evaluation of device performance in the low- and medium-precision application fields, but could not be used to predict the long-term stability or drift in areas requiring high precision, because the polymers actually exhibit viscoelasticity presenting elastic and viscous characteristics simultaneously [11,12], and they also possess the features of time and temperature dependence. The viscoelasticity-related issue has become one of the most critical steps for assessing the packaging quality and output performance of highly precise MEMS sensors [13,14]. Applying the viscoelastic property to model the MEMS devices could give a better agreement with the results observed in experiments than the previous elastic model [15]. The packaging stress in the MEMS was influenced not only by the temperature change, but also by its change rate due to the time-dependent property of polymer adhesives [16]. Besides, the viscoelastic behavior influenced by moisture was recognized as the cause of the long-term stability of micro-sensors in storage [17,18]. Current works on the viscoelastic effects mainly focus on the viscoelasticity-induced reliability issues of MEMS sensors, but the underlying mechanism of stress development over time and temperature is still open to further investigation.

This study aims to deeply investigate the effects of viscoelastic behavior of epoxy-based adhesive on the long-term stability of micro-capacitive accelerometers packaged in a ceramic shell by the adhesively bonding method. Section 2 gives an introduction to the working principle of micro-accelerometers, and theoretically determines the zero offset and sensitivity by the deformation of the sensitive structure of the accelerometer. In Section 3, the accurate viscoelastic property of the adhesive is obtained by a series of stress–relaxation tests by dynamic mechanical analysis (DMA), and the master curve is acquired by the proper shift function and fitted with the Prony function to implement finite element analysis (FEA). In Section 4, the obtained curve is introduced into the physical model of the accelerometer to study the deformation law of sensitive components subjected to four different time and temperature cycles, and then the zero offset and sensitivity of the accelerometer can be determined by the deformed structure. The simulation and experiments are also given in this section. The discussion and conclusion are provided in Section 5 and Section 6, respectively.

## 2. Structure and Principle

**Figure 1:**
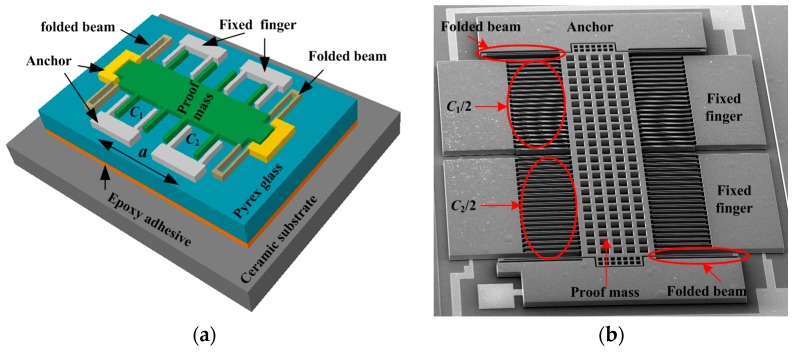
Structure of capacitive accelerometer. (**a**) Diagram of accelerometer; (**b**) SEM structure.

The simplified model of the capacitive accelerometers is shown in Figure 1. The sensitive component is a silicon-based structure supported elastically by four folded beams and bonded to a Pyrex glass substrate. The whole structure is packaged onto a ceramic shell with an epoxy-based adhesive through a curing process. When an acceleration, *a*, is applied in the sensitive direction, the movement of the proof mass induced by inertial force generates a gap change of sensing capacitors formed by a fixed finger on the silicon substrate and a movable finger on the proof mass [19,20]. The changed gap shifts the differential capacitance of capacitors, *C*_1_ and *C*_2_, and the output of the micro-accelerometer can be simply expressed as:

(1) $$V_{out}\;=\;G\frac{C_{1}\;-\;C_{2}}{C_{1}\;+\;C_{2}}V_{b}$$

where *V_out_* is the output voltage of accelerometer, *G* is the amplification coefficient of the sensing circuit and *V_b_* is the amptitude of carrier voltage.

**Figure 2:**
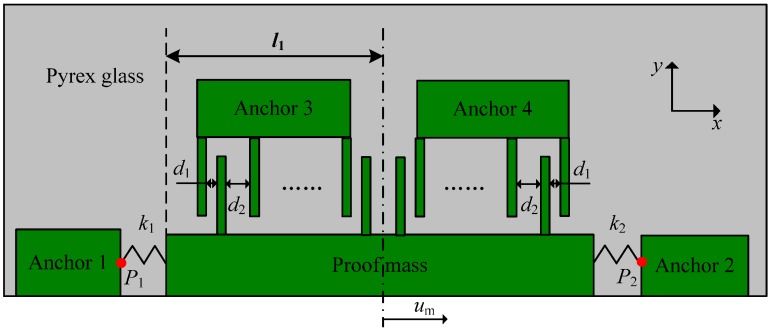
Half-structure of sensitive components.

Half of the sensitive component is selected to derive the analytical expressions of performance, zero offset and sensitivity of the micro-accelerometer. The folded beams anchored to separated sides are represented by two springs with different stiffness constants, *k*_1_ and *k*_2_, due to fabrication errors [21] (Figure 2). The output of the accelerometer, according to Equation (1), is directly proportional to the differential capacitance, which is dependent on the relative position between the fixed fingers and movable fingers. Therefore, the positions of finger anchors (3, 4) and the proof mass determine the performance parameters of the micro-accelerometer, and are influenced by the stress state of the whole structure. Ideally, the free state of packaged accelerometers should show no internal stress, but the packaging process with a fluctuating temperature leads to thermal stress in the chip due to different thermo–mechanical properties of the structural materials. Thus, the zero offset or bias of the micro-accelerometer can be expressed as [22]:

(2) $$Bias=-\frac{Ku_{m}}{m}$$

where *K* denotes the total stiffness of the folded beams, *K* = *k*_1_ + *k*_2_, *m* is the proof mass and *u*_m_ is the displacement of the proof mass center. Furthermore,

(3) $$u_{m}\;=\;\frac{k_{1}\;-\;k_{2}}{k_{1}\;+\;k_{2}}(P_{1x}\;-\;l_{1}\Delta T\alpha_{\mathrm{silicon}})$$

where *P*_1*x*_ denotes the longitudinal displacements of the point connecting the folded beams and the anchors; *l*_1_ is half the length of the proof mass; ∆*T* is the temperature change between curing temperature and room temperature; and *α*_silicon_ is the CTE of silicon.

The sensitivity of the micro-accelerometer can be expressed as:

(4) $$Sensitivity\;=\;(\frac{1}{d_{1}}\;-\;\frac{1}{d_{2}})\frac{Gm}{K}$$

where *d*_1_ and *d*_2_ denote narrow and wide gaps once the curing process is completed, respectively. The sensitivity is mainly determined by the narrow gap, because *d*_1_ is much smaller than *d*_2_.

The Equations (3) and (4) represent the zero offset and sensitivity of the packaged micro-accelerometer, respectively. Both parameters are constant when the structure has no deformation or movement, because the silicon, glass, circuit and internal environment are almost invariant. The micro-accelerometer after storage, however, exhibited an obvious drift of performance parameters. That is to say, the structural equilibrium sustained by the residual thermal stress was upset by some factors related to the structural material properties.

## 3. Material Property

The sensor of the micro-accelerometer consists of four different materials including silicon, Pyrex glass, ceramic material and packaging adhesive. Silicon, Pyrex glass and the ceramic material were used broadly and have very stable material properties with linear elasticity. The Poisson ratio and CTE of the adhesive were assumed as constant values, because they have little impact on the packaging stress [23]. The material properties are listed in Table 1.

**Figure 3:**
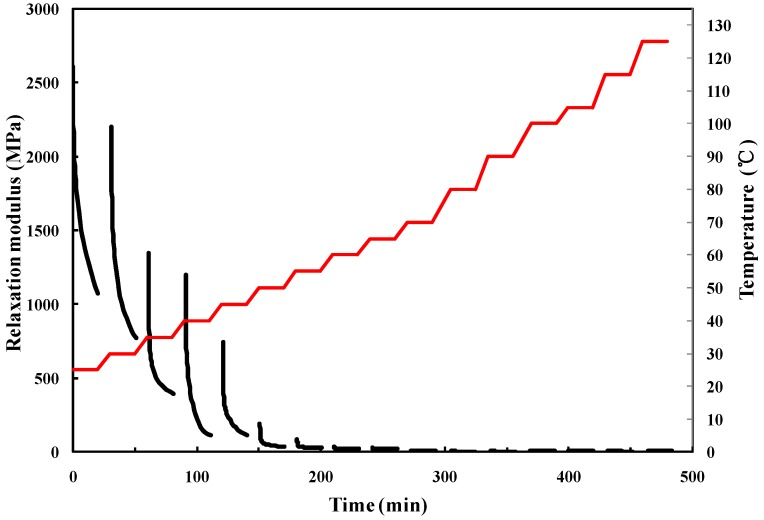
Stress–relaxation test results.

The packaging adhesive, a typical epoxy-based polymer, however, exhibits both elastic and viscous behaviors, that is, viscoelasticity. The elasticity responding to stress is instantaneous, while the viscous response is time-dependent and varies with temperature, which consequently has significant impact on the stress distribution in the micro-accelerometer. To measure the viscoelastic characteristics, therefore, is one of most critical steps before evaluating the stability of micro-accelerometers because of the time- and temperature-dependent feature of the adhesive. The common measuring method is through stress relaxation or creep tests [24]. Hence, a series of stress–relaxation tests were performed using dynamic mechanical analysis (TA instrument DMA Q800, New Castle, DE, USA). A rectangular specimen (35 mm × 10 mm × 2 mm) was prepared and cured by the same process as in the accelerometers, and then mounted on the instrument operating in a single cantilever mode. The experimental temperature range was set from 25 to 125 °C with an increment of 10 °C and an increase rate of 5 °C/min, and at each test point, 5 min was allowed for temperature stabilization and 0.1% strain was applied on the specimen for 20 min, followed by a 10 min recovery. The test results are shown in Figure 3.

The relaxation data, for normalization, were modeled by the master curve, which translates the curve segments at different temperatures to a reference temperature with logarithmic coordinates according to a time–temperature superposition [25]. The master curve can be fitted by a third-order polynomial function, such as:

(5) $$loga_{T}(T)\;=\;C_{1}(T\;-\;T_{0})\;+\;C_{2}{\left(T\;-\;T_{0}\right)}^{2}\;+\;C_{3}{\left(T\;-\;T_{0}\right)}^{3}$$

where *a*_T_ are the offset values at different temperatures (*T*_1_) and *C*_1_, *C*_2_ and *C*_3_ are constants.

**Figure 4:**
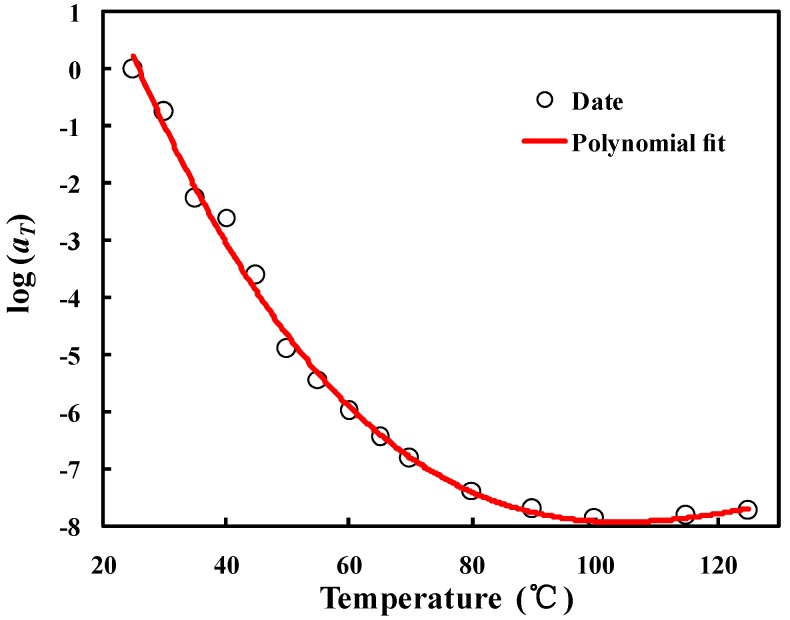
Master curve with reference temperature of 25 °C.

The reference temperature was set at 25 °C, and then the three coefficients of the polynomial function were determined to be *C*_1_ = 0.223439, *C*_2_ = −0.00211 and *C*_3_ = 5.31163 × 10^−6^ (Figure 4).

## 4. Simulation and Experiments

The master curve in Figure 4 shows the relaxation behaviors of polymer adhesives under certain strain loads. This relaxed strain or stress results from the effective modulus of viscoelasticity, which can be described as a Prony series [26]:

(6) $$E(t)\;=\;\frac{\sigma (t)}{\varepsilon_{0}}\;=\;E_{\infty }\;+\;\sum_{i=1}^{N}E_{i}\exp(-\frac{t}{\tau_{i}}),\;\tau_{i}\;=\;\frac{\eta_{i}}{E_{i}}$$

where *E*(*t*) is the relaxation modulus; *σ*(*t*) is the stress; *ε*_0_ is the imposed constant strain; and *E*_∞_ is the fully relaxed modulus. *E_i_* and *τ_i_* are referred to as a Prony pair; *E_i_* is the elastic modulus; *η_i_* is the viscosity; and *τ_i_* is the relaxation time of *i*th Prony pair. *N* is the number of Prony pairs.

**Figure 5:**
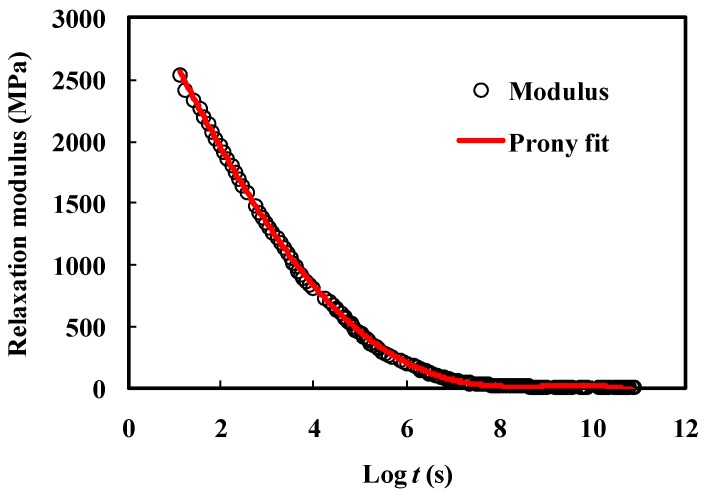
Prony series fitted to master curve.

For this study, the master curve needs to be transformed into Prony series for modeling and experiments (Figure 5). The coefficients of Equation (6) with nine Prony pairs are listed in Table 2, where *E*_0_ is the instantaneous modulus when time is zero.

**Figure 6:**
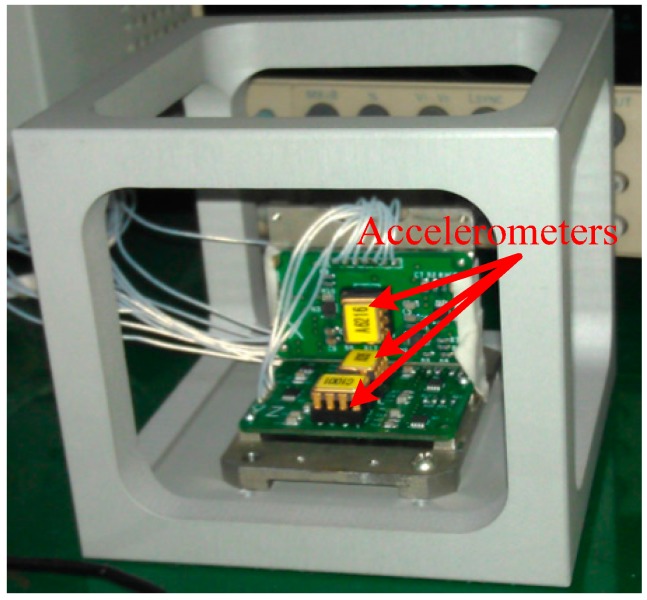
Mounted accelerometers in the test.

**Figure 7:**
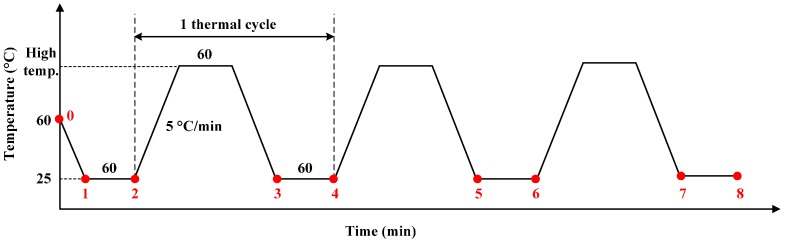
Loading history for the analysis.

The simulation introduced the Prony series modulus into the whole finite element model (FEM) in ABAQUS software to acquire the output of the micro-accelerometers over time and temperature. The thermal experiment was carried out in an incubator with an accurate temperature controller. Each thermal test involved three micro-accelerometers mounted at a cube fixture (Figure 6). The full loading history used in both simulation and experiment is shown in Figure 7. The red-marked points represent the starting or ending points of a loading step. The analysis started at the curing temperature (60 °C, 0) at which the internal stress of the structure is zero. Then, the model was cooled to room temperature (25 °C, 1) with a rate of −5 °C/min and kept at that temperature for 60 min (2), and then the temperature was raised to a higher temperature with 60 min holding, followed by a temperature decrease to room temperature (3) with the same change rate and holding (4). The high temperature points were 50, 75 and 125 °C for the three groups of tests, respectively. The accelerometers in each group were subjected to three high–low temperature steps. The devices kept at room temperature after curing and cooling were also simulated and tested.

**Figure 8:**
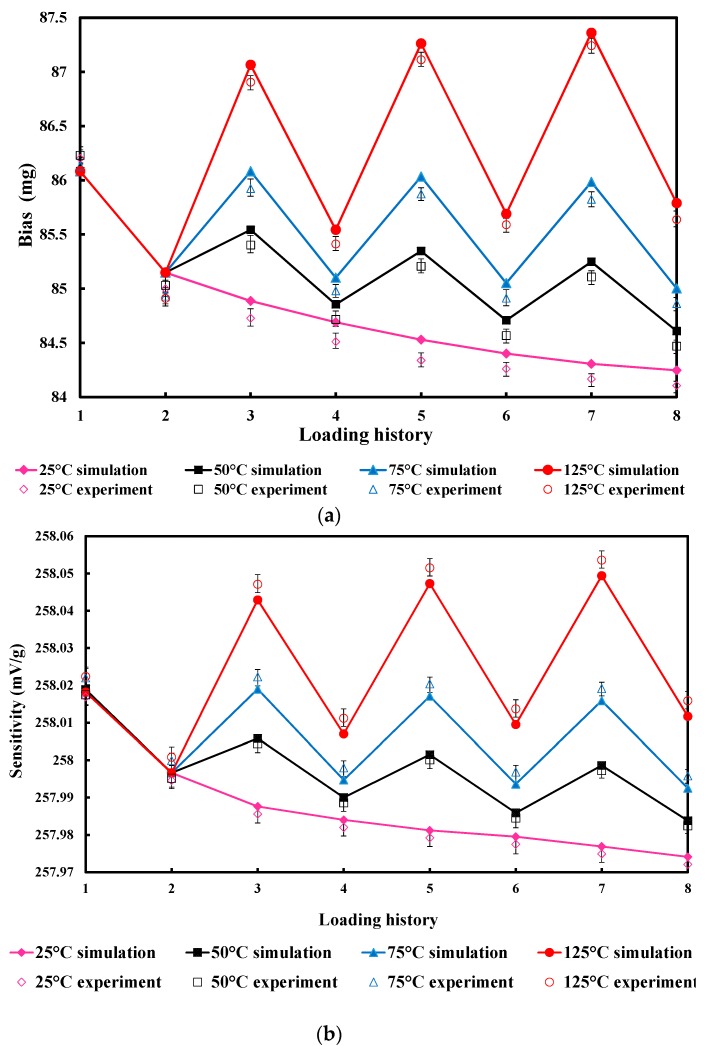
Results comparison. (**a**) Bias; (**b**) sensitivity.

To investigate the time and temperature dependence of device performance, 40 FEM and packaged accelerometers were simulated and tested over the loading history of Figure 7, where the high temperature points were set to 50, 70 and 125 °C. Each thermal cycle was applied to ten accelerometers, and the remaining ten were kept at 25 °C for comparison. The zero offset and sensitivity of the accelerometers are shown in Figure 8. This shows great agreement between experimental and simulation data with small deviation, which is mainly caused by fabrication errors and temperature fluctuation, because the experimental data are the average value of each ten sensors’ results. Both the zero offset and sensitivity of the micro-accelerometers stored at room temperature decreased gradually over time, due to the thermal stress relaxation induced by the viscoelasticity of the polymer adhesive. For the 50 and 75 °C thermal cycles, higher temperature led to a higher offset and sensitivity, and the data of room temperature in each cycle also showed a decrease of offset and sensitivity. However, the 125 °C condition exhibited an increase of offset and sensitivity tested at room temperature.

## 5. Discussion

The observed output drift in the simulation and experiments indicates that the viscoelasticity of adhesive was the main cause of the deviation of zero offset and sensitivity. The underlying mechanism can be attributed to the time- and temperature-dependent stress and deformation states of the sensitive components of the micro-accelerometers. The residual compressive stress and tensile stress existed in the glass and the ceramic substrate, respectively, and because the CTE of ceramic material is larger than glass, the top and bottom interfaces of the adhesive layer exhibit different stress statuses. When the sensors were stored at room temperature, the residual stress in the adhesive gradually changed due to its relaxation induced by the viscoelastic property, and the stress in the chip experienced a corresponding shift to influence the deformation of sensitive components.

**Figure 9:**
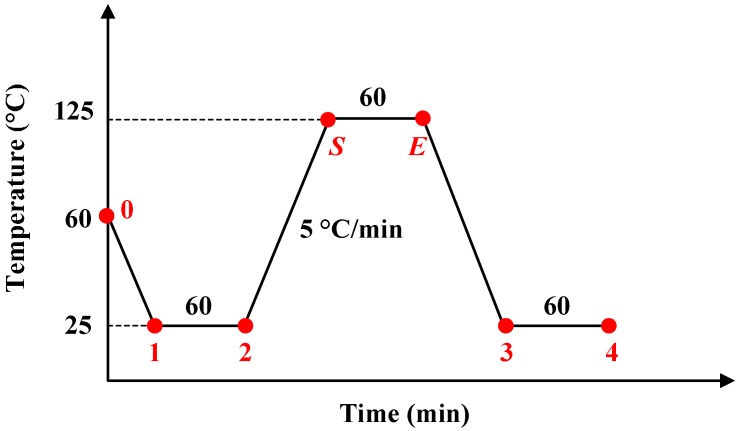
The first thermal cycle process at 125 °C.

**Figure 10:**
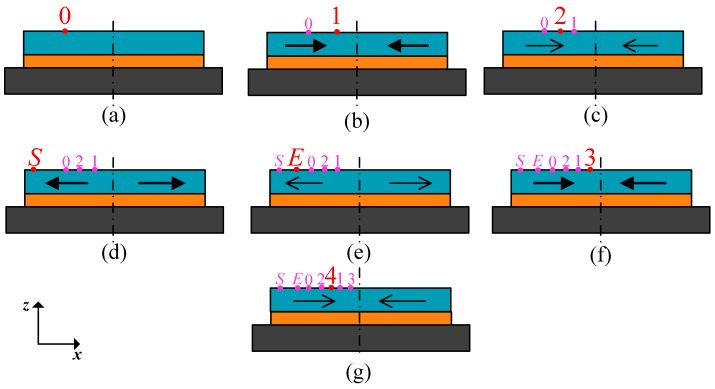
The moving trail of point *P*_1_ at each step in the first thermal cycle at 125 °C. (**a**) Curing process; (**b**) cooling to room temperature; (**c**) room temperature retention; (**d**) starting point of high temperature; (**e**) ending point of high temperature; (**f**) cooling to room temperature again; (**g**) room temperature retention again.

The stress relaxation of the adhesive at room temperature resulted in an expansion of the glass layer because of the compressive stress in it, so the distance of points *P*_1_ and *P*_2_ was extended (Figure 2), which led to a broadened gap *d*_1_ and a reduced displacement of proof mass based on Equation (3). The sensitivity and zero offset, therefore, decreased over time. In the thermal cycle experiments, the stress state in the chip changes with the loading process, including time and temperature effects. Take the experiment at 125 °C as an example; the key points of temperature change are marked in Figure 9. The displacement of point *P*_1_ is selected to explain the structural deformation of the sensitive components over time, and the temperature during heating and cooling processes (Figure 10). The detailed information can be concluded in the following steps:

- Step 1—curing process: The initial state or stress-free state of the sensor appeared at 60 °C where the curing process started, and the *P*_1_ was located at position 0 in Figure 10a.
- Step 2—cooling to room temperature: The point *P*_1_ moved to the center, the proof mass generated a positive movement along *x* direction and gap *d*_1_ decreased (Figure 10b). Thus, the offset and sensitivity both increased at this stage compared with those in Figure 10a.
- Step 3—room temperature retention: The compression of glass decreased due to stress relaxation, and *P*_1_ slightly moved in the negative *x* direction, which led to the decrease of *u*_m_ and increase of *d*_1_ (Figure 10c). The offset and sensitivity decreased.
- Step 4—starting point of high temperature: The model expanded, and the point *P*_1_ moved in the negative *x* direction substantially, which caused the decrease of *u*_m_ and increase of *d*_1_ (Figure 10d). Therefore, the offset and sensitivity continued to decrease.
- Step 5—ending point of high temperature: During the thermal treatment process, the tension on the glass decreased due to stress relaxation, and *P*_1_ slightly moved in the positive *x* direction to result in a higher offset and sensitivity (Figure 10e).
- Step 6—cooling to room temperature again: The model shrank, and the shrinkage included both the thermal part in step 4 and additional shrinkage value generated by strong viscoelasticity at 125 °C. Hence, the point *P*_1_ moved to the right position 1 (Figure 10f). Consequently, the offset and sensitivity at this stage were larger than those of Figure 10b.
- Step 7—room temperature retention again: The offset and sensitivity decreased, due to stress relaxation, but they were still larger than those in Figure 10c. This indicated that the offset level is influenced by thermal treatment, so higher-temperature dwells lead to greater offset and sensitivity (Figure 10g).

For the accelerometers exposed to thermal cycles of 50 and 75 °C, the displacement pattern of point *P*_1_ is the same as that of 125 °C, but with a smaller value, because of the smaller expansion of structures.

## 6. Conclusions

The viscoelasticity effects of the epoxy-based die attach adhesive on the output performance of MEMS capacitive accelerometers over time and temperature were studied by simulation and experimental methods. The zero offset and sensitivity gradually decreased due to stress relaxation when accelerometers were kept at room temperature after adhesive packaging. In the same period, the high-temperature load slowed down the process of stress relaxation, and was not advantageous in improving the long-term stability related to viscoelasticity of packaging adhesives. Furthermore, the drift of bias and sensitivity can be attributed to the residual stress development induced by the viscoelastic behavior of adhesion materials. To aid in an in-depth understanding of the viscoelastic behavior of materials, future work will concentrate on developing the theoretical model of viscoelasticity of materials in the tri-layered assembly. Furthermore, the viscoelasticity difference induced by preparation and process will be studied to find out the solution to the drift of micro-devices.

## Figures and Tables

**Table 1:** Material properties for the sensor.

Material	Young’s Modulus (Gpa)	Poisson Ratio	CTE (ppm/°C)
Silicon	160	0.22	2.6
Glass	62.7	0.2	3.25
Ceramic	360	0.22	6.5
Adhesive	Table 2	0.37	60

**Table 2:** Prony pairs of the die attach adhesive.

*i*	*E_i_*/*E*_0_^1^	$\tau_{i}$
1	0.08510	3041.87694
2	0.14589	981,765.85865
3	0.22654	243.32699
4	0.11248	2083.25650
5	0.15906	48,993.21024
6	0.21617	31.16988
7	0.03923	5052.23180
8	0.00025	6992.77554
9	0.00676	3321.33054

^1^ *E*_0_ = 2744.76252 MPa.
